# Improved estimates of coordinate error for molecular replacement

**DOI:** 10.1107/S0907444913023512

**Published:** 2013-10-12

**Authors:** Robert D. Oeffner, Gábor Bunkóczi, Airlie J. McCoy, Randy J. Read

**Affiliations:** a Cambridge Institute for Medical Research, University of Cambridge, Hills Road, Cambridge CB2 0XY, England

**Keywords:** *Phaser*, maximum likelihood, molecular replacement

## Abstract

The estimate of the root-mean-square deviation (r.m.s.d.) in coordinates between the model and the target is an essential parameter for calibrating likelihood functions for molecular replacement (MR). Good estimates of the r.m.s.d. lead to good estimates of the variance term in the likelihood functions, which increases signal to noise and hence success rates in the MR search. *Phaser* has hitherto used an estimate of the r.m.s.d. that only depends on the sequence identity between the model and target and which was not optimized for the MR likelihood functions. Variance-refinement functionality was added to *Phaser* to enable determination of the effective r.m.s.d. that optimized the log-likelihood gain (LLG) for a correct MR solution. Variance refinement was subsequently performed on a database of over 21 000 MR problems that sampled a range of sequence identities, protein sizes and protein fold classes. Success was monitored using the translation-function *Z*-score (TFZ), where a TFZ of 8 or over for the top peak was found to be a reliable indicator that MR had succeeded for these cases with one molecule in the asymmetric unit. Good estimates of the r.m.s.d. are correlated with the sequence identity and the protein size. A new estimate of the r.m.s.d. that uses these two parameters in a function optimized to fit the mean of the refined variance is implemented in *Phaser* and improves MR outcomes. Perturbing the initial estimate of the r.m.s.d. from the mean of the distribution in steps of standard deviations of the distribution further increases MR success rates.

## Abstract 2

A function for estimating the effective root-mean-square deviation in coordinates between two proteins has been developed that depends on both the sequence identity and the size of the protein and is optimized for use with molecular replacement in *Phaser*. A top peak translation-function *Z*-score of over 8 is found to be a reliable metric of when molecular replacement has succeeded.

## 1. Introduction

Molecular replacement (MR; Rossmann & Blow, 1962 ▶) relies on the evolutionary principle that two proteins with a high sequence identity are very likely to have similar secondary and tertiary structures and hence low root-mean-square deviation (r.m.s.d.) in coordinate positions. An estimate of the r.m.s.d. is an essential parameter used to calibrate likelihood functions in the maximum-likelihood approach to MR (Read, 2001 ▶). If the estimate is good, then appropriate weight is placed on the agreement of reflections at different resolutions and it is not necessary to apply arbitrary resolution cutoffs. However, if the estimate is poor then the signal is reduced and a correct solution may not be detectable in the MR search.

The r.m.s.d. is introduced into the likelihood targets *via* the parameter σ_A_, 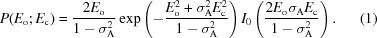
σ_A_ is a function of resolution (measured by *s* = 1/*d*, the absolute value of the diffraction vector) that combines the effects of positional errors of the atoms in the model (the r.m.s.d.) and the completeness of the model *f*_P_, *i.e.* the ratio between the scattering power of the model and of the crystal (Read, 1986 ▶; Srinivasan & Ramachandran, 1966 ▶). Ignoring the effects of bulk solvent, σ_A_ can be expressed in the simple form given in (2), 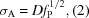
where *D* = exp[(−2π^2^/3)*s* ^2^r.m.s.d.^2^] and *f*_P_ = Σ_P_/Σ_N_.

To account for defects in the model associated with the lack of bulk solvent, a low-resolution falloff is also incorporated into the equation for σ_A_,
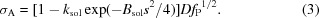
When an MR calculation is undertaken within the maximum-likelihood formalism, σ_A_ is initialized from estimates of r.m.s.d. and *f*_P_, typically using generic values for *k*_sol_ and *B*_sol_ (McCoy *et al.*, 2007 ▶). If the r.m.s.d. is underestimated σ_A_ will be overestimated and the log-likelihood gain (LLG) will be smaller than with the correct r.m.s.d. Similarly, an overestimate of r.m.s.d. leads to an underestimate of σ_A_ and again a reduction in the LLG.

Prior to successful molecular replacement, only the sequence of the target is available to inform the estimation of an appropriate r.m.s.d. value. Chothia & Lesk (1986 ▶) formulated an expression for the relationship between sequence identity and r.m.s.d. in main-chain atoms based on 32 pairs of homologous structures,
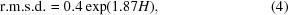
where *H* is the fraction of mutated residues between the two sequences. At a sequence identity of 100%, (4) has a minimum of 0.4 Å. Experiences with a number of test cases (data not shown) indicated that this value was frequently too low for the estimate of the variance term in the maximum-likelihood functions as implemented in *Phaser* (McCoy *et al.*, 2007 ▶), leading to negative LLG scores, and therefore the formula used in *Phaser* was modified with a lower bound of 0.8 Å, which applied in effect above 63% sequence identity. The r.m.s.d. estimated for the purpose of calculating the variance used in the likelihood function in *Phaser* (e.r.m.s.d.) was taken as
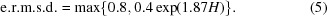
After the model has been correctly placed, it is possible to refine the r.m.s.d. parameter that determines the σ_A_ values by maximizing the LLG. We term this optimized r.m.s.d. parameter the variance-RMS (VRMS). We anticipated that (4) was suboptimal for estimating the VRMS for four reasons. Firstly, the equation was derived from a very small database of only 32 structures and they represented a narrow range of comparative lengths of between only 99 and 287 residues. Since the publication of (4) in 1986, the PDB has expanded to include more than 90 000 structures of up to 1500 residues, all of which are potential models for MR. Secondly, unlike the r.m.s.d., the VRMS is not biased by any explicit atom-pair assignment. Thirdly, the actual r.m.s.d. is not necessarily the best effective VRMS to use in the equation for σ_A_; the r.m.s.d. continues to grow dramatically as the errors grow, whereas structure-factor agreement does not become worse once the error is comparable to the *d*-spacing. Fourthly, we are interested in the best effective VRMS to use for the subset of cases for which an MR solution can be found; in the low-identity range in particular this will bias VRMS to lower values corresponding to models that are better than average. We aimed to find a better initial estimate of VRMS from the information available prior to structure solution, namely the sequence identity to the target, the number of residues in the model and the fold class. For these reasons, an estimate for the VRMS cannot be directly equated with an r.m.s.d. computed from a structural alignment between two structures. Even if it were possible to obtain a structure-based r.m.s.d. prior to solving the structure, this r.m.s.d. would not be as useful as the VRMS value that maximizes the likelihood in an MR calculation. By the same token, it would be incorrect to employ the VRMS for situations in which a structure-based r.m.s.d. value is required.

## 2. Methods

A database of 21 822 MR calculations was generated for optimizing the estimation of the VRMS. Computations were performed on an Ubuntu 64-bit queueing-system cluster with five dual-processor quad-core nodes and a total of 320 Gb of memory.

### 2.1. Target structures

2862 structures were selected from the PDB using the criteria that they were biological monomers, that they had one monomer in the asymmetric unit and that the associated X-ray data had been deposited. Twinned structures were excluded, as were structures for which the published *R* factor could not be reproduced.

**Figure 1:**
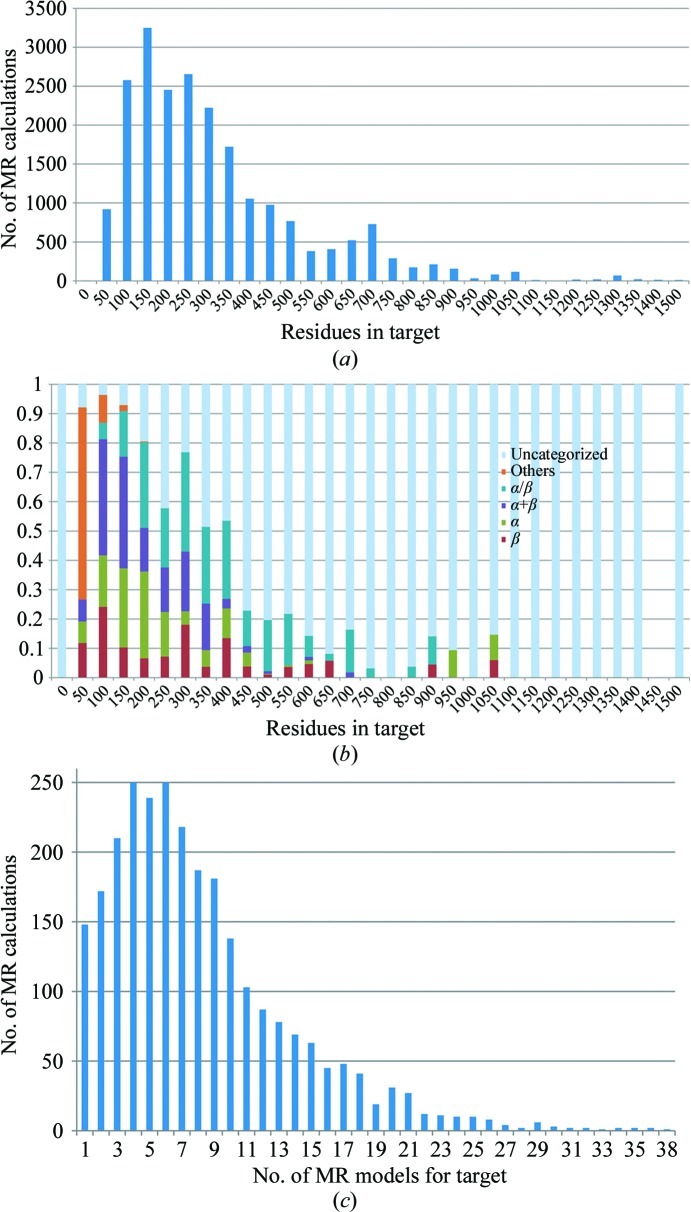
(*a*) Number of MR calculations as a function of the number of residues in their respective MR targets. (*b*) Fraction of MR calculations with a target belonging to certain SCOP classes as a function of the number of residues in the target. (*c*) Histogram of the number of MR models used in MR calculations.

The number of entries in the PDB varies drastically across the range of protein sizes from very small (fewer than 50 residues) to large (more than 1000 residues). The vast majority of proteins are in the moderate-size range of between 100 and 500 residues. Targets were chosen across the range of sizes in the PDB. All PDB structures with 600 residues or more that met the selection criteria were retained, but nonetheless the relatively small number of large structures available limited the quality of the statistics for the largest proteins. The distribution of sizes used is shown in Fig. 1 ▶(*a*).

Targets were chosen across the range of SCOP classes (Murzin *et al.*, 1995 ▶). There are ten SCOP classes, of which we focused only on the four main classes: ‘all-alpha (α)’, ‘all-beta (β)’, ‘alpha and beta proteins (α+β)’ and ‘alpha and beta proteins (α/β)’. The current SCOP database, from 23 February 2009, annotates 38 221 PDB entries. This is about half of the number of PDB entries as of the commencement of this study and so a significant fraction of the target structures was uncategorized. The number of proteins belonging to the SCOP classes varies according to the number of residues in the protein (Fig. 1 ▶ *b*). Very small proteins of 50 or fewer residues do not belong to any of the four SCOP classes under consideration. Proteins in the moderate-size range are uniformly distributed across the SCOP classes.

### 2.2. Model structures

A *BLAST* search (Altschul & Lipman, 1990 ▶) for homologous PDB structures was performed using each target sequence. The searches were performed using an in-house *BLAST* server with a local copy of the nonredundant PDB. The *BLAST* searches used the *BLASTP* algorithm with the BLOSUM62 matrix. To ensure that all matches between sequences were recorded, the number of sequences to show alignments for was set to 20 000 and the expectation value was set to a large value (1000). The *BLAST* algorithm works by scoring local alignments (*i.e.* subsequences) between structures and gives higher sequence identities than global alignments. Sequence identities were therefore recalculated with *ClustalW* (Thompson *et al.*, 1994 ▶), which maximizes global sequence alignment. The sequence identity was taken as the fraction of identical residues in the total alignment length. Sequences with sequence identities below 15% and above 60% were excluded. This is the range of sequence identity that is of interest for this study, since MR rarely fails at identities above 60% and MR rarely succeeds at identities below 15%. The structures corresponding to these PDB entries were pruned and edited with *Sculptor* (Bunkóczi & Read, 2011 ▶) using the default protocol. On average, eight MR models were found per target. The composition of the database with regard to the number of models per target is shown in Fig. 1 ▶(*c*).

### 2.3. Templates

For each model and target pair, a transformation to superimpose the model onto the target was determined. An initial superposition with *SSM* (Krissinel & Henrick, 2004 ▶) was followed by rigid-body refinement with *Phaser* to find the six-­dimensional global LLG maximum. Potential solutions obtained from MR were analysed with respect to this transformation, accounting for symmetry operations and allowed origin shifts, to identify the correct solutions.

## 3. Results

A total of 21 822 MR calculations were analysed to find those that succeeded and those that failed. The translation-function *Z*-score (TFZ) for the top peak in the search was found to be a reliable indicator of successful MR, at least for this class of cases in which there is one molecule in the asymmetric unit. *Z*-­scores measure the number of standard deviations over the mean. The mean and standard deviation for the translation-function search were taken from a random sample of 500 positions of the model in the same orientation. Note that there can be additional incorrect peaks in a translation search that are lower than the top peak but still with a nonrandom TFZ. These usually arise from solutions that are partially correct, such as translations that place a molecule correctly relative to one symmetry axis but not relative to perpendicular axes; such solutions give a better than random prediction of the data.

**Figure 2:**
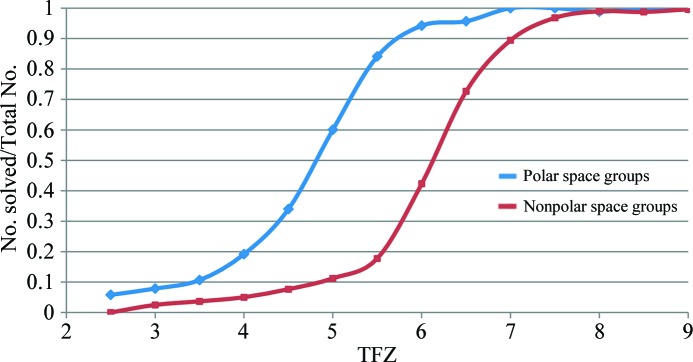
Fraction of correct placements of the only/first component in the asymmetric unit as a function of TFZ by polar and nonpolar space group. Polar space groups accounted for one quarter of the test cases in our database, while the 1% of test cases that were in space group *P*1 were excluded from this analysis.

The placement of the only/first model in polar space groups is ambiguous in the direction of the polar axis. In space group *P*1 the placement of the first/only model is redundant. In nonpolar space groups a peak TFZ of 8 or more indicated a successful solution, while in polar space groups a peak TFZ of 6 was sufficient. Approximately half of the solutions with a TFZ of 6.5 were correct in nonpolar space groups. While correct solutions could be found with TFZ values as low as 5, they were not necessarily the top peak and it was not clear *a priori* that these solutions were correct. The ratio of correct to the total number of solutions by TFZ is shown in Fig. 2 ▶.

We anticipate that the top TFZ criterion will also apply to searches for subsequent components, which will be tested in future studies. However, it should be noted that the presence of translational noncrystallographic symmetry (tNCS) is a complication. If no account is taken of the effect of tNCS, adding a second molecule in the same orientation as the first molecule even in an incorrect solution will give a high LLG and TFZ score for a translation that separates the two molecules by a vector corresponding to the major off-origin peak in the Patterson map. Fortunately, this artefact can be eliminated by a tNCS correction (McCoy & Read, unpublished work) based on a statistical understanding of the effects of tNCS (Read *et al.*, 2013 ▶).

### 3.1. Dependence on sequence identity

**Figure 3:**
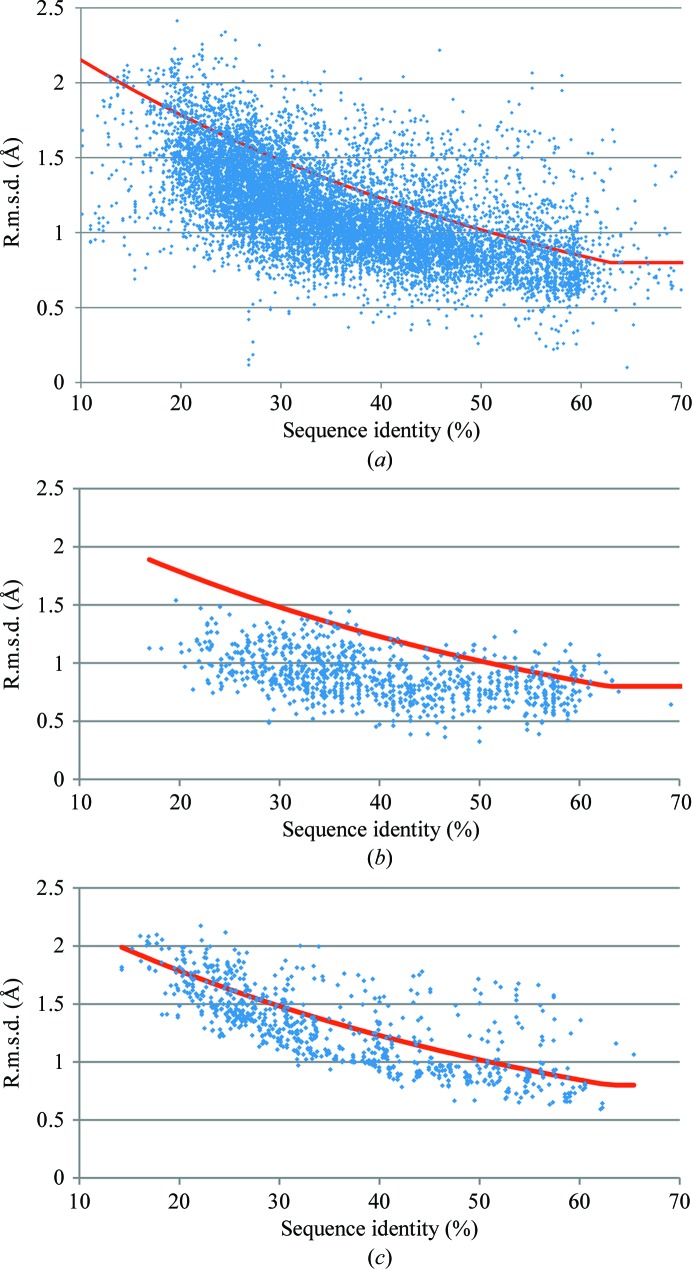
Scatter plot of VRMS against sequence identity for correct MR solutions: 10 921 data points. The red line represents (5) in *Phaser*. (*a*) All data, (*b*) data for models of less than 100 residues, (*c*) data for models of between 400 and 500 residues.

Of the 21 822 MR calculations, 10 921 yielded correct solutions for which VRMS refinement gives useful results for further analysis. Fig. 3 ▶(*a*) shows a scatter plot of VRMS *versus* sequence identity for correct MR solutions. The distribution of the VRMS values deviates significantly from the estimate of e.r.m.s.d. in (5). In general the VRMS is overestimated by (5), particularly at low sequence identities. This can be explained in part by the implicit selection of models that are sufficiently good to succeed in MR for the analysed subset. However, the distribution of refined VRMS about its mean when plotted by sequence identity alone (Fig. 3 ▶ *a*) is broad.

### 3.2. Dependence on number of residues

Figs. 3 ▶(*b*) and 3 ▶(*c*) show scatter plots of VRMS values for the data separated into bins by number of residues. The distribution about the mean value is significantly narrower when the data are binned in this way. It is evident that the more residues in the model, the better the Chothia and Lesk e.r.m.s.d. agrees with the VRMS value. Note that the overall results in Fig. 3 ▶(*a*) are biased towards small structures, which are seen more frequently in the database (Fig. 1 ▶). The number of residues is therefore a significant second variable in the VRMS estimation.

### 3.3. Estimate of VRMS

**Figure 4:**
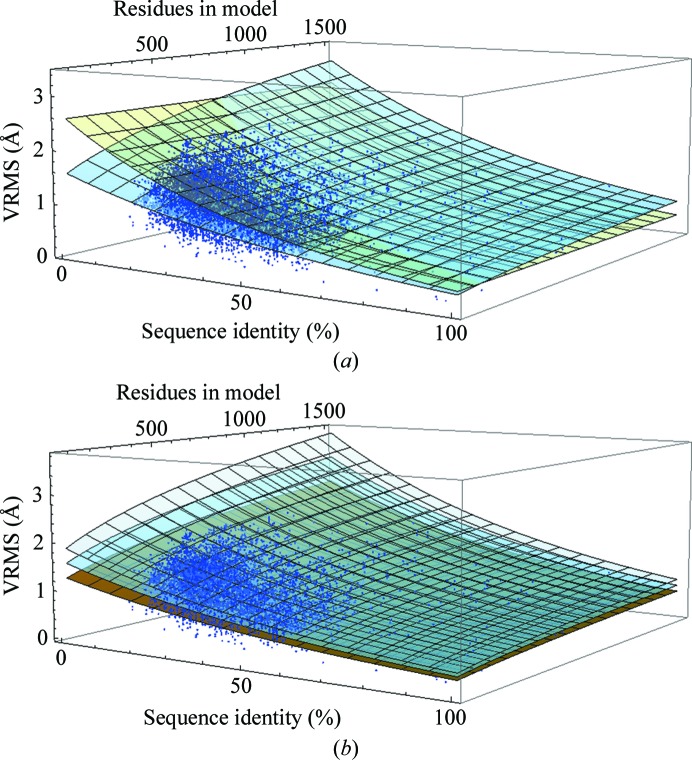
(*a*) Fit of the eVRMS (light blue surface) and the Chothia and Lesk r.m.s.d. in (4) (pale yellow surface) to the refined VRMS values of 10 921 MR solutions. The effective limits of eVRMS (sequence identity, number of residues) are eVRMS (100%, 15) = 0.362 Å and eVRMS (15%, 1500) = 2.53 Å. (*b*) Fit of the eVRMS (light blue surface) and eVRMS ± 1σ surfaces to the refined VRMS values of 10 921 MR solutions.

The functional form of the equation with which to fit the refined VRMS with sequence identity and number of residues as parameters was chosen to fulfil a number of limiting conditions. Firstly, the equation was required to increase monotonically. Secondly, for any particular size of protein (measured by number of residues) the equation was required to adopt the functional form of the Chothia and Lesk formula. Thirdly, the increase in estimated VRMS was made dependent on the overall linear dimensions of a protein by taking the cube root of a linear function of the number of residues in the model, which assumes that proteins have similar shapes. The functional form for the estimated VRMS (eVRMS) was therefore taken as
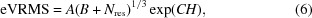
where *N*_res_ is the number of residues in the model and *H* is, as in (5), the fraction of mutated residues. A fit of the parameters *A*, *B* and *C* to the 10 921 VRMS values for the correct MR solutions was carried out in *Mathematica* (Wolfram Research Inc., Champaign, Illinois, USA) and produced *A* = 0.0569, *B* = 173, *C* = 1.52. This constitutes a two-dimensional surface, which is shown in Fig. 4 ▶(*a*). The mean residual of the Chothia and Lesk e.r.m.s.d. to all data points is 0.269 Å, whereas with the fit in (6) it is 0.160 Å. The eVRMS deviates most from the Chothia and Lesk r.m.s.d. at low sequence identity and for proteins of up to 500 residues in length.

In contrast to the earlier implementation of e.r.m.s.d. (5) using the Chothia and Lesk equation (4), we have not applied a lower bound for the eVRMS in (6) for two reasons. Firstly, if the eVRMS estimate is too low the model is still likely to be very good, so that MR will usually succeed, and a negative LLG at the end of MR, previously associated with low initial estimates of the r.m.s.d., is now avoided by VRMS refinement as the final step in MR in *Phaser*. Secondly, the previous lower bound of 0.8 Å was too pessimistic when searching with precise models comprising fewer than 50 residues, such as helices in the *ARCIMBOLDO* procedure (Rodríguez *et al.*, 2009 ▶).

**Figure 5:**
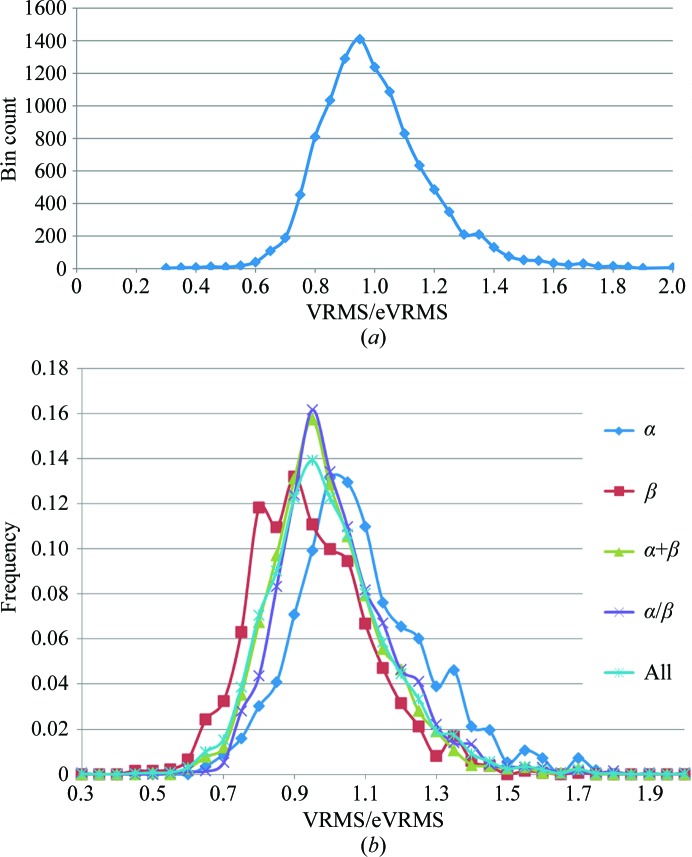
(*a*) Histogram of VRMS/eVRMS for the 10 921 correct solutions in the MR database. The distribution is approximately Gaussian. (*b*) Frequency distribution of VRMS/eVRMS for the four major SCOP classes computed for models ranging from 100 to 300 residues in length.

The significant scatter of VRMS values above and below the eVRMS surface indicates that inflating or deflating the VRMS estimate may be required in difficult cases. To determine the appropriate sampling distance, a histogram of the ratio of VRMS to eVRMS values is shown in Fig. 5 ▶(*a*) based on the assumption that the width of the distribution of VRMS values is proportional to the mean. The histogram is observed to be approximately Gaussian with a standard deviation of σ_(VRMS/eVRMS)_ = 0.1965. This lets us define surfaces in steps of σ_(VRMS/eVRMS)_ from (6) by simple multiplication of the eVRMS by a fractional difference, as illustrated in Fig. 4 ▶(*b*).

### 3.4. Test of the VRMS estimate

To test how well the VRMS estimate in (6) affects the success rate in MR calculations, we re-evaluated a subset consisting of 3375 borderline cases from our MR database using the new r.m.s.d. estimates computed with (6). We define borderline cases as those MR calculations for which the template MR solution yields an LLG value within the interval (20, 90) as well as having a global map correlation between the electron densities of the MR solution and the target of greater than 0.2. MR problems that do not belong to this set almost always pose little challenge to solve (LLG over 90) or have no credible solution at all (LLG below 20 or map correlation below 0.2). Preliminary calculations with the proposed r.m.s.d. estimate showed clear gains in TFZ values for easy MR problems. It is, however, the borderline cases that matter in practice. The TFZ values improved somewhat in calculations that used (6) rather than (5). For this set of calculations we found the average values shown in Table 1 ▶. While the average TFZ increase between the Chothia and Lesk e.r.m.s.d. in (5) and the new eVRMS in (6) appears to be small, it should be remembered that the VRMS values used for the calculation of the eVRMS were not limited to borderline cases only. They also included values for MR calculations in which the correct solutions are found with high TFZ.

σ_(VRMS/eVRMS)_ was used to calibrate the perturbation of the VRMS to sample above and below the eVRMS. In Table 2 ▶ the numbers of solved borderline cases are shown for eVRMS and VRMS estimates perturbed in steps of ±½σ_(VRMS/eVRMS)_ and ±1σ_(VRMS/eVRMS)_. The total number of MR trials that can be solved with at least one of the five estimates is 3036, or 89.8%, of the borderline cases. The number of trials that can be solved with at least one of the five estimates but not with the Chothia and Lesk e.r.m.s.d. is 259, whereas the number of trials that are only solved with the Chothia and Lesk e.r.m.s.d. is 20. An analysis of these 20 cases shows that they are all represented by points that have refined VRMS values well above the eVRMS surface in Fig. 4 ▶(*a*), in the corner (sequence identity <36%, fewer than 280 residues) where the Chothia and Lesk e.r.m.s.d. estimate deviates most from the new estimate. The average eVRMS is 1.15 Å for these 20 cases, while the average refined VRMS of 1.53 Å is identical to the estimate from (5). MR solutions for 12 of these 20 cases can be rescued by extending the exploration of the VRMS to include +1.5σ_(VRMS/eVRMS)_ and a further five by extending it to include +2σ_(VRMS/eVRMS)_. For the three remaining cases the signal in the MR search is very weak even when the search succeeds; in such cases there is a stochastic element to whether or not the correct solution ends up in the reported list of solutions.

When the estimated coordinate error was not perturbed, the best set of results was obtained with the eVRMS values (Table 2 ▶), which failed to yield solutions for only 594 of the test cases. By perturbing the eVRMS with five different estimates, the number of failures was reduced to only 339, which means that about one third of the failed solutions could be rescued.

In these borderline cases where finding the correct solution can depend on using the right VRMS estimate, *Phaser* frequently reports more than one plausible solution with a TFZ less than 8; the correct solution is not necessarily at the top of the list, so it could not be identified with confidence. Nonetheless, these solutions could be used as candidates in the recently developed *MR-Rosetta* procedure (DiMaio *et al.*, 2011 ▶), which has been shown to yield a 50% success rate for further model building based on MR solutions with poor TFZ scores. Likewise, these solutions could also be used as a starting point for the morphing procedure (Terwilliger *et al.*, 2012 ▶).

### 3.5. Dependence on SCOP class

We also investigated the dependence of the VRMS on the SCOP class. Fig. 5 ▶(*b*) shows the distributions of the VRMS/eVRMS values for the four SCOP classes of moderate-sized proteins under consideration in this study. From these distributions we can deduce the means and standard deviations listed in Table 3 ▶.

Proteins belonging to the ‘all-β’ class have a VRMS that is overestimated by about 5% on average, whereas those for ‘all-­α’ proteins are underestimated by about 9% on average. This suggests that the overall folds for proteins dominated by β-­sheets are better conserved than those composed of α-­helices. Apart from the ‘all-α’ class, which is more variable, the standard deviations show that the distributions separated into fold categories are slightly narrower than the total distribution that combines all fold categories. However, this analysis has not been used to further refine estimates of the VRMS based on fold class in *Phaser* because there is still a very large overlap among the distributions for different fold classes compared with the standard deviations of the distributions and hence it is likely that little would be gained compared with sampling the estimates of the VRMS in fractions of σ_(VRMS/eVRMS)_. At the same time, there would be much added complication in determining and passing information about the fold class to *Phaser*.

## 4. Discussion

By using the new eVRMS in (6) instead of the Chothia and Lesk e.r.m.s.d. in (5), we have achieved a better estimate of the r.m.s.d. for use in maximum-likelihood MR. This is partly because of the addition of size dependence, which accounts for the fact that homologous large structures have long-range structural perturbations (for example, twists or small hinge motions) that inflate the r.m.s.d. over the r.m.s.d. commonly found in homologous smaller structures, and partly because the Chothia and Lesk formula was not designed to provide an effective VRMS for MR calculations. The new eVRMS increases the success rate with *Phaser* for borderline MR problems. This is therefore now the default setting in *Phaser* for estimating the VRMS for an MR model with respect to the unknown target structure.

The new eVRMS provides a good overall fit to the mean of the refined VRMS values, but there is significant spread about the mean. In cases in which a clear solution is not found using the estimated eVRMS, additional trials should be carried out using higher and lower estimated values consistent with the observed spread. Our database of test cases also enabled us to estimate the standard deviation of this spread about the mean and hence useful sampling distances above and below the mean. Such a procedure would rescue cases in which the MR search failed with the new r.m.s.d. values but succeeded with the previous Chothia and Lesk e.r.m.s.d. estimates. An option to inflate or deflate the default r.m.s.d. estimate by 1σ above and below the mean has been implemented in *Phaser*, but a broader and finer exploration of this parameter could increase success in pipelines, particularly when following MR with automated rebuilding tools.

To determine the sequence identity we used *ClustalW*, in part because this is a tool that is readily available to users of *Phaser*. One might expect that more sophisticated tools such as *HHpred* (Söding *et al.*, 2005 ▶) would yield more precise estimates of the sequence identity between structurally aligned residues. However, a control experiment (results not shown) demonstrated that this is unlikely to yield improvements in the quality of the eVRMS estimates. We repeated the curve-fitting of the VRMS as a function of sequence identity and model size but using sequence identities obtained by structural alignment, and found that the proportional error in the eVRMS estimates was equivalent to that obtained using *ClustalW* alignments.

We have followed Chothia and Lesk in basing the estimated r.m.s.d. on sequence identity, largely because this is an easy parameter for users of *Phaser* to provide. Nonetheless, there could be advantages to using more subtle measures of sequence similarity. Below 30% sequence identity, it has been shown that the expectation values produced by tools such as *BLAST* are better correlated than the sequence identity with the r.m.s.d. value between structures (Wilson *et al.*, 2000 ▶). Incorporating such a measure instead of, or in addition to, the sequence identity may be valuable for improving the eVRMS estimates in future work.

## 5. Availability

All methods described are implemented in *Phaser* 2.5.4. *Phaser* is available through the *CCP*4 (http://www.ccp4.ac.uk; Winn *et al.*, 2011 ▶) and *PHENIX* (http://www.phenix-online.org; Adams *et al.*, 2002 ▶) software distributions. *Phaser* documentation can be found at http://www.phaser.cimr.cam.ac.uk.

## Figures and Tables

**Table 1:** Average translation-function *Z*-scores (TFZ) for 3375 cases for the VRMS estimates derived from the Chothia and Lesk e.r.m.s.d. as given by (5) and the eVRMS given by (6) and perturbed by σ_(VRMS/eVRMS)_ values, where eVRMS_±*n*σ_ = eVRMS[1 ± *n*σ_(VRMS/VRMS)_]

Chothia and Lesk e.r.m.s.d.	eVRMS_−1σ_	eVRMS_−½σ_	eVRMS	eVRMS_+½σ_	eVRMS_+1σ_
〈TFZ〉 = 6.28	〈TFZ〉 = 6.37	〈TFZ〉 = 6.47	〈TFZ〉 = 6.48	〈TFZ〉 = 6.43	〈TFZ〉 = 6.34

**Table 2:** Matrix of results from 3375 borderline cases solved with the five different estimates of the VRMS against cases not solved with the five different estimates, where eVRMS_±*n*σ_ = eVRMS[1 ± *n*σ_(VRMS/VRMS)_] Diagonal elements are the total number of solved calculations of the borderline cases with a particular estimate. Off-diagonal values are the number of calculations solved with the *i*th estimate (row) that cannot be solved with the *j*th estimate (column).

	eVRMS_+1σ_	eVRMS_+½σ_	eVRMS	eVRMS_−½σ_	eVRMS_−1σ_	Chothia and Lesk e.r.m.s.d.
eVRMS_+1σ_	2840	80	123	139	151	63
eVRMS_+½σ_	57	2863	74	95	111	81
eVRMS	92	66	2871	64	85	82
eVRMS_−½σ_	122	101	78	2857	45	133
eVRMS_−1σ_	171	154	136	82	2820	182
Chothia and Lesk e.r.m.s.d.	105	146	155	192	204	2798

**Table 3:** Mean and standard deviation of the ratio of VRMS to eVRMS as a function of SCOP class The results for the total four SCOP classes only include proteins for which a SCOP class was assigned.

	All-α	All-β	α+β	α/β	Total four SCOP classes
VRMS/eVRMS	1.089	0.946	0.990	1.019	0.997
σ_(VRMS/eVRMS)_	0.187	0.167	0.157	0.168	0.172
